# Directly visualizing the sign change of *d*-wave superconducting gap in Bi_2_Sr_2_CaCu_2_O_8+δ_ by phase-referenced quasiparticle interference

**DOI:** 10.1038/s41467-019-09340-5

**Published:** 2019-04-08

**Authors:** Qiangqiang Gu, Siyuan Wan, Qingkun Tang, Zengyi Du, Huan Yang, Qiang-Hua Wang, Ruidan Zhong, Jinsheng Wen, G. D. Gu, Hai-Hu Wen

**Affiliations:** 1 National Laboratory of Solid State Microstructures and Department of Physics, Center for Superconducting Physics and Materials, Collaborative Innovation Center for Advanced Microstructures, Nanjing University, 210093 Nanjing, China; 2 Condensed Matter Physics and Materials Science Department, Brookhaven National Laboratory, Upton, New York, 11973 USA

## Abstract

The superconducting state is formed by the condensation of Cooper pairs and protected by the superconducting gap. The pairing interaction between the two electrons of a Cooper pair determines the gap function. Thus, it is pivotal to detect the gap structure for understanding the mechanism of superconductivity. In cuprate superconductors, it has been well established that the gap may have a *d*-wave function. This gap function has an alternative sign change in the momentum space. It is however hard to visualize this sign change. Here we report the measurements of scanning tunneling spectroscopy in Bi_2_Sr_2_CaCu_2_O_8+δ_ and conduct the analysis of phase-referenced quasiparticle interference (QPI). We see the seven basic scattering vectors that connect the octet ends of the banana-shaped contour of Fermi surface. The phase-referenced QPI clearly visualizes the sign change of the *d*-wave gap. Our results illustrate an effective way for determining the sign change of unconventional superconductors.

## Abstract (web summary)

The superconducting gap structure contains important information to understand the pairing mechanism of unconventional superconductivity. Here, by using a newly established phase-referenced quasiparticle interference technique, the authors visualize the sign change of the *d*-wave superconducting gap directly in Bi_2_Sr_2_CaCu_2_O_8+δ_.

## Introduction

In superconductors, the charge carriers are Cooper pairs which carry the elementary charge of 2*e*. This can be easily concluded from the measurements of the quantized flux *Φ*_0_ = *h*/2*e* = 2.07 × 10^−15^ Wb based on the Ginzburg–Landau theory. The core issue and also the most hard problem for understanding the mechanism of superconductivity is how the two conduction electrons are bound to each other to form a pair, the so-called Cooper pair. In the theory of Bardeen-Cooper-Schrieffer (BCS), it was predicted that the Cooper pairs in some superconductors, mainly in elementary metals and alloys, are formed by exchanging the virtue vibrations of the atomic lattice, namely phonons. The two electrons in the original paired state (**k**, *−***k**) will be scattered to another paired state (**k**′, *−***k**′). This scattering will lead to the attractive interaction *V*_*k*,*k*′_, and the electron bound state will be formed with the help of suitable Coulomb screening. Condensation of these electron pairs will lead to superconductivity. This condensate is protected by an energy gap *Δ*(*k*) which prevents the breaking of Cooper pairs. Usually the gap is a momentum dependent function which is closely related to the pairing interaction function *V*_*k*,*k*′_. For example, in above mentioned pair scattering picture, one can easily derive the function

1 $${\it{\Delta}} ( k ) = - \mathop {\sum }\limits_{k\prime } V_{k,k\prime }\frac{{\it{\Delta}} ( k\prime )}{2E( k\prime )}{\mathrm{tanh}}\frac{E ( k\prime )}{2k_{\mathrm{B}}T}.$$

Here, $E\left( k \right) = \sqrt {\varepsilon ^2 + {\it{\Delta}} ^2(k)}$ with *ε* the kinetic energy of the quasiparticles counting from the Fermi energy *E*_F_. If this pairing process can apply to other unconventional superconductors, the sign of the gap *Δ*(*k*) would change if the pairing interaction *V*_*k*,*k*′_ is positive.

For cuprate superconductors, it has been well documented that the gap has a *d*-wave form *Δ* = *Δ*_0_cos2*θ*. This basic form of the gap was first observed by experiments of angle resolved photo-emission spectroscopy (ARPES) without sign signature^1,2^, and later supported by many other experiments, such as thermal conductivity^3^, specific heat^4,5^, scanning tunneling microscopy (STM)^6–8^, neutron scattering^9,10^, and Raman scattering^11^, etc. Although some of the techniques mentioned above may involve the sign change of the gap, such as the inelastic neutron scattering and STM measurements, they cannot tell how the gap sign changes explicitly along the Fermi surface in the momentum space.

In the model system of cuprate superconductor Bi_2_Sr_2_CaCu_2_O_8+δ_ (Bi-2212), the low energy excitations have been measured very carefully by STM yielding the seven scattering vectors or spots in the Fourier transformed (FT-) quasi-particle interference (QPI) pattern^12,13^. These seven scattering vectors were explained very well with the Fermi arc picture as evidenced by ARPES measurements^14^. Combining the magnetic effect on the QPI intensities of the different vectors, Hanaguri et al.^15^ showed the well consistency of a *d*-wave gap, if assuming that the magnetic vortices behave like magnetic scattering centers and that leads to the enhancement (suppression) of the joint QPI intensity of two momenta (**k**_1_, **k**_2_) with the gaps of the same (opposite) signs. The sign problem of a *d*-wave gap was also resolved by the experimental devices based on the Josephson effect^16–18^. Therefore *d*-wave superconducting gap has been well concluded in cuprate superconductors by using many experimental tools.

In this paper, we report the experiments and analysis based on the defect-induced bound state (DBS-) QPI method^19,20^ on Bi-2212 by concerning the referenced phases at positive and negative energies. Actually in conventional superconductor 2*H*-NbSe_2_ with magnetic impurities, and anti-phase feature of the bound states at positive and negative energies were found^21^. The authors successfully interpreted this effect as the phase difference of the Shiba function at positive and negative biased energies. Our results illustrate the direct visualization of the sign change of the *d*-wave gap in bulk Bi-2212.

## Results

### Topography and tunneling spectra measured on Bi-2212

**Fig. 1:**
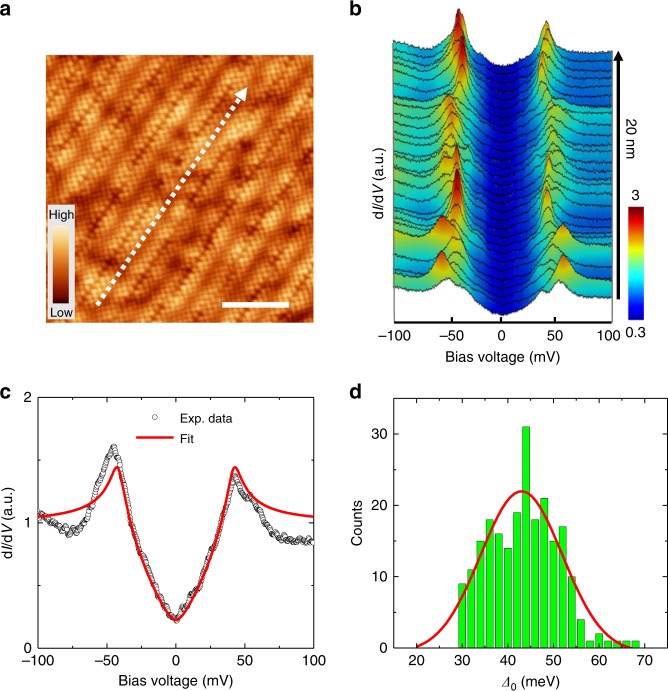
Topographic image and tunneling spectra measured on optimally doped Bi-2212. **a** A typical topographic image of the BiO surface after cleavage measured at bias voltage *V*_bias_ = 50 mV and tunneling current *I*_t_ = 100 pA. Scale bar, 5 nm. **b** Tunneling spectra measured along the arrowed line in **a**. The set-point conditions are *V*_set_ = 100 mV and *I*_set_ = 100 pA for measuring all the spectra in **b**. **c** A typical spectrum (open circles) selected from **b**. The solid line shows the Dynes model fitting result with a *d*-wave superconducting gap. **d** The statistics of superconducting gap maxima *Δ*_0_ for about 200 tunneling spectra measured on the Bi-2212 samples, and the gap maximum values are determined from the positions of the coherence-peaks. The red curve is a Gaussian fitting result with the peak position near 43 meV

Figure 1a shows a typical topographic image of the BiO surface of Bi-2212 with an atomic resolution after cleavage. The supermodulations in the real space can be clearly witnessed on the surface with a period of about 2.54 nm. We can also recognize a square lattice structure with the lattice constant *a*_0_ about 3.8 Å, even with the appearance of the supermodulations. In Fig. 1b, we present a sequence of tunneling spectra measured along the arrowed line in Fig. 1a. The spectra show the V-shaped bottoms near zero bias, which reveals the intrinsic feature of the gap nodes in optimally doped Bi-2212. One can also clearly see the spatial variation of the coherence-peak positions, being consistent with previous reports^6,7,22^. We then chose one typical spectrum in Fig. 1b and plot it in Fig. 1c. The finite zero-bias differential conductance implies the effect of the impurity scattering^6,7,23^ in the case of a nodal gap. We then use the Dynes model^24^ with a *d*-wave gap function *Δ*(*θ*) = *Δ*_0_ cos2*θ* to fit the tunneling spectrum, and the fitting result is also shown in Fig. 1c by the red line. The resultant fitting yields the parameters of gap maximum *Δ*_0_ = 42 meV, and the scattering rate *Γ* = 4 meV. One can see that the fitting curve with the *d*-wave gap function captures the major characteristics except for the feature of the coherence peak on the negative-bias side. It should be noted that the gap maximum equals approximately to the coherence-peak energy in a *d*-wave superconductor when the scattering rate is small. Following this conclusion, we carry a statistical analysis of the gap maximum values from the coherence-peak energies of about 200 spectra measured in our experiments on optimally doped Bi-2212 samples, and the related histogram of the gap distribution is presented in Fig. 1d. One can see that the distribution of *Δ*_0_ behaves as a Gaussian shape with an averaged gap maximum $\bar{\it{\Delta}} _0 = 43\,{\mathrm{meV}}$ obtained from the fitting.

### Identification of scattering wave-vectors in FT-QPI pattern

**Fig. 2:**
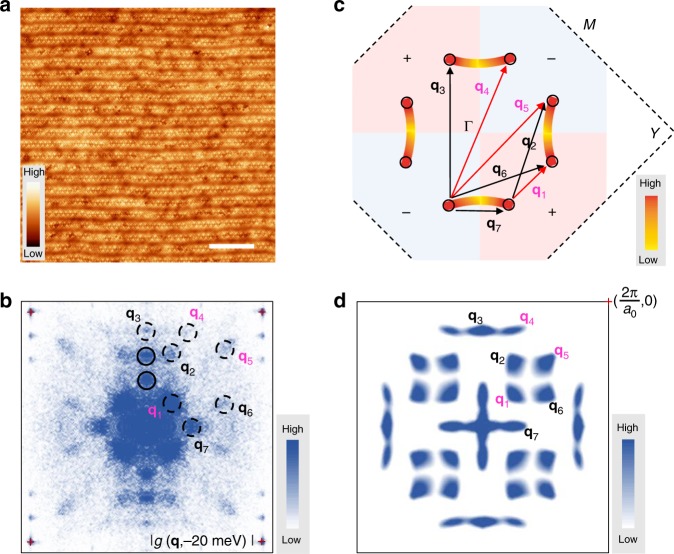
Different kinds of scattering wave-vectors in FT-QPI pattern. **a** Topographic image of the BiO plane (*V*_bias_ = −200 mV, *I*_t_ = 50 pA). Scale bar, 10 nm. **b** FT-QPI pattern (*V*_set_ = −100 mV, *I*_set_ = 100 pA) at *E* = −20 meV based on the QPI image measured in the area of **a**. Four Bragg peak positions are marked by the red crosses. The solid-line circles indicate the scattering spots contributed by the supermodulations, while the dashed circles indicate the primary scattering spots with different **q** vectors. **c** A schematic plot of the contours of constant energy. The DOS along the Fermi surface is set to be **k**-dependent, and the intensities at the octet ends of CCE are the strongest. The different colors of light pink and light blue show the regions with different gap signs for the *d*-wave gap function. The scatterings along black arrows (**q**_2_, **q**_3_, **q**_6_, and **q**_7_) are for sign reversal gaps, while the scatterings along magenta arrows (**q**_1_, **q**_4_, and **q**_5_) are sign-preserved ones. **d** The simulation of FT-QPI by applying the self-correlation to **c**

Figure 2a shows a topographic image with the dimensions of 56 × 56 nm. In this area, we measured the QPI images *g*(**r**, *E*) at different energies, and then obtained the FT-QPI pattern *g*(**q**, *E*) in **q**-space through the Fourier transform. Figure 2b shows a typical FT-QPI pattern at –20 meV. Since we have done the lattice correction process by using the Lawler–Fujita algorithm^25^, the four Bragg peaks are very sharp near the red crosses marked in Fig. 2b. Based on the octet model^12^, the octet ends of the banana-shaped contour of constant energy (CCE) has relatively higher density of states (DOS). Therefore, the major scattering will occur among these hot spots. As expected, we can clearly distinguish all the primary scattering wave vectors **q**_*i*_ (*i* = 1 to 7) indicated by dashed circles in the FT-QPI pattern. Besides, two additional pairs of spots, which are indicated by solid-line circles, are originated from the supermodulations^12^. It should be noted that the **q**_7_ spots at the horizontal direction are not influenced by the supermodulations. Figure 2c shows a schematic plot of the contours at a particular energy below the superconducting gap maximum in a typical cuprate superconductor like Bi-2212, and the DOS at the octet ends are set to be maximum. By assuming a certain width for the Fermi arcs in Fig. 2c, we then apply the self-correlation and show the simulated FT-QPI result in Fig. 2d. One can see that the simulated patterns are similar to those in the experimental FT-QPI pattern, which can help identify different primary scattering wave vectors. In a *d*-wave superconductor, the superconducting gap changes its sign in **k**-space along the Fermi surfaces, and we use different background colors to show this sign-change feature in Fig. 2c. With the aid of light pink and light blue colors for different gap signs, we can divide the scattering wave vectors into two groups, i.e., gap-sign-reversed scatterings (with scattering wave vectors of **q**_2_, **q**_3_, **q**_6_, and **q**_7_) and gap-sign-preserved ones (**q**_1_, **q**_4_, and **q**_5_).

### Energy evolution for different scattering vectors

**Fig. 3:**
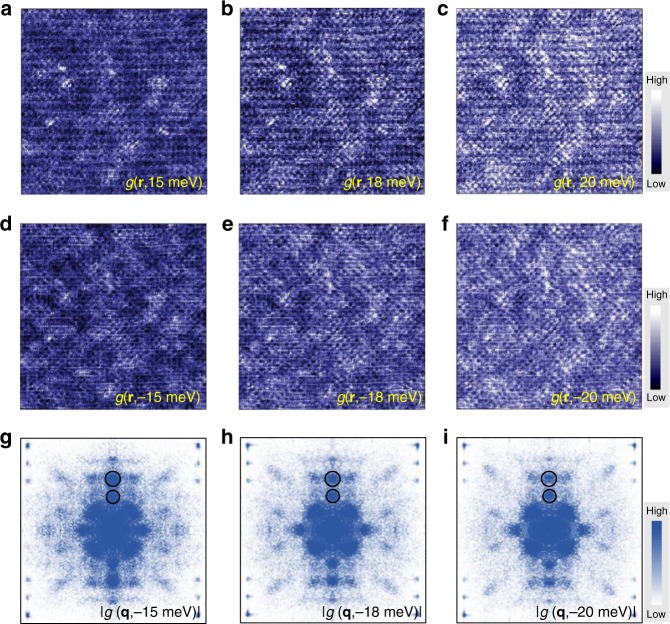
QPI and FT-QPI patterns measured on Bi-2212. **a**–**f** Differential conductance mappings at different energies. **g**–**i** Three corresponding FT-QPI patterns of **d**–**f**, respectively. The supermodulations contribute to the FT-QPI intensity surrounded by the solid-line circles, and these spots seem to have very weak energy dispersion

It is known that the Bogoliubov quasiparticles will be scattered by impurities and the induced standing waves will interfere with each other, giving rise to the Friedel-like oscillations of local DOS (LDOS). Figure 3a–f present the QPI images measured at different energies. We can see clear standing waves in these QPI images, which are due to the scattering by some impurities or defects in the sample such as randomly distributed oxygen deficiencies^23^. The longitudinal periodic modulations as shown in the topography of Fig. 2a mainly contribute the spots enclosed by the solid-line circles along *q*_*y*_ in Fig. 2b. While the other spots in Fig.2b should be contributed by the scatterings between the octet ends of CCE. We then do the Fourier transform to QPI images and show them for three selected energies in Fig. 3g–i, and the measured QPI images together with the corresponding FT-QPI patterns at other energies are shown in Supplementary Fig. 1. One can clearly recognize the scattering spots from the supermodulations and seven characteristic scattering channels in the FT-QPI patterns. It should be noted that the coordinates of supermodulation spots in FT-QPI patterns are non-dispersive in energy, which can also be taken as a justification for the origin of the supermodulation. However, the **q***-*vectors of seven characteristic scattering spots seem to be dispersive, which can be naturally illustrated by the octet model^12^.

**Fig. 4:**
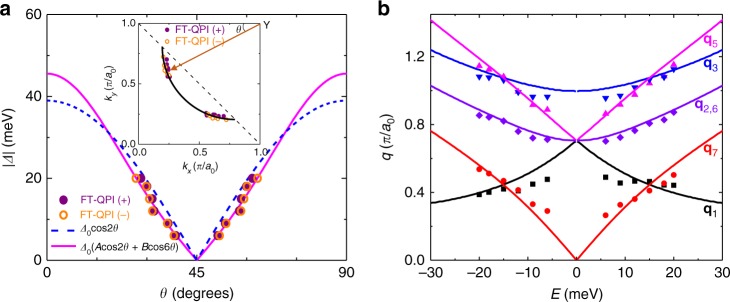
Energy dispersions for characteristic scattering wave vectors and superconducting gap. **a** Superconducting gap *Δ*(*θ*) (circles) and two fitting curves by different *d*-wave gap functions. The inset in **a** shows the positions of the ends of CCE determined by **q**_1_ and **q**_7_ measured at various energies. The solid line shows the Fermi surface from the fitting to these positions by a circular arc, and it is cut off by the dashed line. The angle *θ* for each end of CCE is defined in the inset. The gap value *Δ* for each end of CCE is equivalent to the energy at which the QPI data are measured. **b** Energy dispersion of the scattering wave vectors taken from the FT-QPI data (excluding **q**_4_ due to very weak intensity). The solid lines represent the theoretical predictions based on the Fermi surface and the *d*-wave gap function with high order shown by the line with magenta color in **a**

To obtain the information of superconducting gaps, we then analyze the energy dispersions of the characteristic scattering wave vectors. The intensities of the spots corresponding to **q**_1_ and **q**_7_ scattering vectors are stronger than the spots corresponding to other scattering vectors, and the central coordinates of these spots can be obtained by fitting to a two-dimensional Lorentzian function^13^. In principle, these two scattering wave vectors can provide enough information to fix the coordinates of the octet ends of CCE at various energies within the superconducting gap maximum. The resultant positions of the ends of CCE are shown in the inset of Fig. 4a, and the data allow us to construct part of the Fermi surface. The solid curve in the inset is a fitting result to the positions of the ends of CCE by a circular arc. The curve, which is intentionally cut off by the dashed line^26^, represents the contour of the Fermi surface of Bi-2212 at this doping level. From the inset of Fig. 4a, we can also define the angle of these ends of CCE to the (0, π) to (π, π) direction. With the combination of the defined angle *θ* and the measured energies as the absolute values of superconducting gaps at the ends of CCE, we can obtain the angular dependent superconducting gap *Δ*(*θ*) along the Fermi surface and show it in Fig. 4a. The obtained *Δ*(*θ*) data are fitted by two different *d*-wave gap functions, i.e., *Δ*_1_(*θ*) = 39cos2*θ* meV and *Δ*_2_(*θ*) = 45.6 (0.9cos2*θ* + 0.1cos6*θ*) meV, and the latter shows a better consistence with the experimental data. Therefore, the gap function in optimally doped Bi-2212 may have a higher order in addition to the *d*-wave component, which is consistent with previous report^12^. Looking back to other scattering spots, the intensity of **q**_4_ scattering spot is very weak, thus the dispersion associated with **q**_4_ scattering spot is not shown here. The positions of **q**_2_ and **q**_6_ scatterings are equivalent with only the exchange of *k*_*x*_ and *k*_*y*_ coordinates. Thus, we focus only on five sets of characteristic scattering spots with the center coordinates obtained by the fittings to two-dimensional Lorentzian functions. The obtained energy dispersions of these characteristic wave vectors are presented in Fig. 4b, being consistent with previous reports^8,12,22^. The solid curves in Fig. 4b represent the predicted energy dispersions based on the Fermi surface in the inset of Fig. 4a and the gap function *Δ*_2_(*θ*). One can see that the calculated curves agree well with the experimental data, which indicates the validity of the octet model and the *d*-wave superconducting gap.

### Theoretical approach of using the DBS-QPI method

The PR-QPI method was first theoretically proposed by Hirschfeld, Altenfeld, Eremin, and Mazin in a two-gap superconductor^27^, which was successfully applied to prove the gap-sign-change in iron-based superconductors for a non-magnetic impurity^28,29^. The recently proposed DBS-QPI method^19,20^ is designed for judging the gap-sign issue in iron-based superconductor LiFeAs around a non-magnetic impurity, and is also successfully used to confirm the gap sign-change in (Li_1−*x*_Fe_*x*_)OHFe_1−*y*_Zn_*y*_Se from our recent work^30^. In this method, the QPI image *g*(**r**, *E*) is measured in an area with a non-magnetic impurity sitting at the center of the field of view (FOV). The FT-QPI data, which comes from the Fourier transform of *g*(**r**, *E*), are complex parameters containing the phase information, namely $g\left( {{\mathbf{q}},E} \right) = \left| {g\left( {{\mathbf{q}},E} \right)} \right|e^{i\varphi _g\left( {{\mathbf{q}},E} \right)}$. Then the phase-referenced (PR-) QPI signal can be extracted from the phase difference between positive and negative bound state energies, that is defined by

2 $$g_{\mathrm{r}} \left( {{\mathbf{q}}, + E} \right) = \left| {g\left( {{\mathbf{q}}, + E} \right)} \right|,$$

3 $$g_{\mathrm{r}}\left( {{\mathbf{q}}, - E} \right) = \left| {g\left( {{\mathbf{q}}, - E} \right)} \right|{\mathrm{cos}}(\varphi _{{\mathbf{q}}, - E} - \varphi _{{\mathbf{q}}, + E}).$$

Here, *g*_r_(**q**, +*E*) should be always positive by the definition, and *g*_r_(**q**, −*E*) should be negative near the bound state energy for the scattering involving the sign-reversal gaps at **k**_1_ and **k**_2_ (**q** = **k**_1_ − **k**_2_). That conclusion was drawn by the simulation for a nodeless superconductor when the gap changes its sign for different Fermi pockets in LiFeAs (refs. ^19,20^). Our studies in (Li_1−*x*_Fe_*x*_)OHFe_1−*y*_Zn_*y*_Se reveal that this method can also work for concentric two-circle like Fermi surfaces when the gaps on them have opposite signs^30^.

In cuprates, the gap value varies continuously and gap nodes appear in the nodal direction (Γ − Υ) on the Fermi surface. The FT-QPI patterns have already been calculated in several previous works on cuprate systems^31–37^. However, it is very curious to check whether this phase-referenced DBS-QPI method is still applicable in a *d*-wave superconductor. Before checking, it seems that there are no bound state peaks on the tunneling spectrum in our present sample, but nevertheless the DBS-QPI technique can still be applicable. In cuprates, the intrinsic nanoscale electronic disorders such as oxygen vacancies or crystal defects can act as the scattering centers, which will influence the tunneling spectra^23^. According to the previous calculation^38^, if the scattering potential is small, the bound state peaks are absent in a *d*-wave superconductor. To further elaborate this issue, we do the theoretical calculations by a standard T-matrix method^31^ with the details described in Method part. We use a *d*-wave gap in the calculation, and the calculated angle-dependent superconducting gap and Fermi surface in Supplementary Fig. 2a are consistent with the experimental data. Supplementary Fig. 2b shows the tunneling spectra at an impurity-free area and on site of the non-magnetic impurity with scattering potential *V*_s_ = 20 meV. One can see that there are no obvious in-gap bound state peaks near zero-bias; the non-magnetic impurities only induce resonance state and produce a continuous change of DOS within the superconducting gap when the scattering potential is small. The further calculated spectra are shown in Supplementary Figs. 3 and 4 with different scattering potentials. One can see that the clear resonance peaks can be observed only when |*V*_s_| is much larger than 100 meV. Otherwise, there are only asymmetric intensity change or some small humps within the gap. The simulation results following the DBS-QPI method for a single impurity are shown in Supplementary Figs. 2–4. One can see that the PR-QPI signal for the gap-sign-preserved scatterings (**q**_1_, **q**_4_, and **q**_5_) are all positive, while the signal for the gap-sign-reversed ones (**q**_2_, **q**_3_, **q**_6_, and **q**_7_) are all negative, which gives a sharp contrast. Concerning the gap sign change issue, the simulated results in a *d*-wave superconductor are consistent with the situation in the *s*^±^ superconductor LiFeAs (refs. ^19,20^), and this validates our calculation method.

### Multi-DBS-QPI method applied on experimental data in Bi-2212

As shown in the line-scan spectra in Fig. 1b, it seems there are no sharp bound state peaks at energies below 30 meV, however, we do have observed the QPI images reflecting the seven characteristic spots. This tells that the standing waves on the QPI images are obviously induced by the widely distributed non-magnetic impurities which lead to resonance states instead of sharp bound state peaks in Bi-2212. For better illustrating this point, we have conducted several other line-scan measurements in the sample and presented the results in Supplementary Fig. 5. One can see that, in all three line-scan spectroscopies, although the tunneling spectra are relatively homogenous at low energies, small kinks or humps are however observed on many of the spectra at the energies from 10 to 30 meV. In addition, all the spectra show sizable magnitude of DOS near Fermi energy. This is certainly induced by pair breaking effect from impurity scattering. Combining the theoretical calculations and our experimental results, we believe that the resonance states do exist on the spectra and are induced by widely distributed non-magnetic impurities with relatively weak scattering potentials. Thus, it is difficult to find the exact impurity locations. Given the absence of the bound state peaks, we still try to analyze the measured QPI data to obtain the information of gap sign by using the DBS-QPI method.

**Fig. 5:**
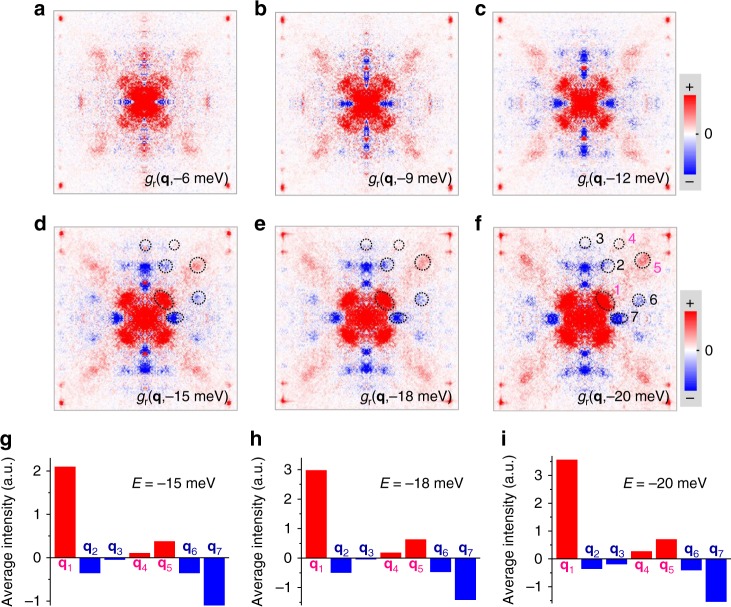
PR-QPI signal by multi-DBS-QPI method. **a**–**f** PR-QPI images obtained directly from the FT-QPI patterns measured at different energies. Each digit in **f** denotes the position of each characteristic scattering wave-vectors. **g**–**i** Averaged intensity per pixel of PR-QPI signal for each **q** at −15, −18, and −20 meV, respectively. The areas for the integral and average are marked by dashed circles or ellipses in **d**–**f**

Figure 5a–f show the PR-QPI patterns at different negative energies. The PR-QPI patterns at positive energies are not shown here, because all the signals should be positive without any extra phase information according to Eq. (2). At energies from −6 to −12 meV, the *g*_r_(**q**,−*E*) signals of **q**_**1**_ and **q**_**7**_ are clear and easily recognized. When the energy is lowered down to below −15 meV, all the seven characteristic scattering spots become even more clear and can also be easily recognized. We then calculate the average value to the signals in the areas within the dashed-circles or ellipses in Fig. 5d–f, and the histograms of the average intensities per pixel corresponding to different scattering channels are shown in Fig. 5g–i. The signals corresponding to **q**_1_, **q**_4_ and **q**_5_ spots are positive, while those corresponding to **q**_2_, **q**_3_, **q**_6_ and **q**_7_ spots are negative. According to the logic possessed by the DBS-QPI method, we naturally argue that those **k** points in the momentum space connected by **q**_1_, **q**_4_ or **q**_5_ have sign-preserved superconducting gaps, and those connected by **q**_2_, **q**_3_, **q**_6_ or **q**_7_ have sign-reversed ones. This conclusion is consistent very well with our theoretical calculation above by using a *d*-wave gap function. Since the theoretical model used for the Fermi surface is rather rough, it is thus reasonable to see different intensities of the corresponding spots of the PR-QPI signal between the experimental data (Fig. 5g–i) and the calculated results (Supplementary Figs. 2–4), which will be further addressed below. In order to show the validity of this conclusion, we have done some control experiments on other two samples in three different areas, and the results are presented in Supplementary Figs. 6 and 7. One can see that the new results are well consistent with those presented in Fig. 5, and they show exactly the expected results for a *d*-wave superconducting gap. It should be noted that the sign difference for corresponding scattering spots can be easily recognized at energies below 25 meV; however, the sign cannot be resolved when the energy is above 25 meV (Supplementary Fig. 6). The reason for this is that the characteristic scattering spots themselves become blurred at the energies above 25 meV, and only a central spot is left when the energy is near the superconducting gap (~40 meV). This seems to be a common feature in Bi-2212 samples, which was also reported in previous studies^8,13,22^. The reason is unclear yet, and it could be induced by the involvement of the anti-nodal region which is more associated with the pseudogap^13,39^. This can also explain the spatial variation of the intensity and shape of the coherence peaks in Bi-2212. An alternative picture for this spatial variation of coherence peaks would be the combination of disorder and electron–electron interactions as inferred in two-dimensional electron gas system Pb/Si(111) monolayer film^40^. This requires of course further verification.

**Fig. 6:**
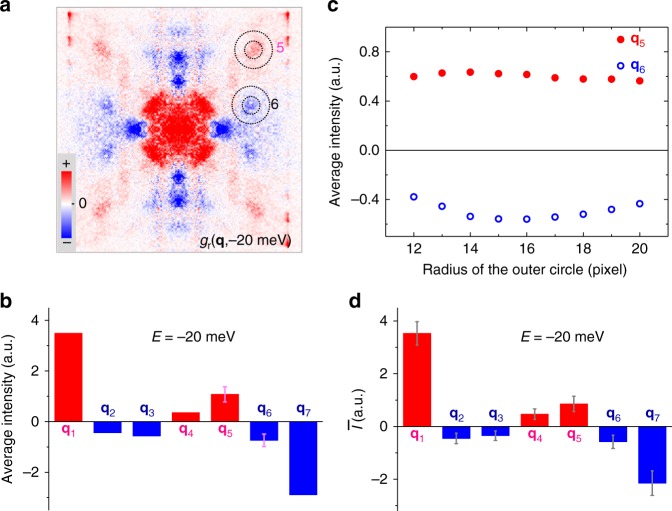
Control experiments and error analysis of the PR-QPI signal. **a** Experimental PR-QPI pattern measured on another sample and analyzed by multi-DBS-QPI method. **b** Averaged intensity per pixel of PR-QPI signal for each scattering spot. **c** The averaged intensity of the PR-QPI signal within the two circles shown in **a**. This calculation for determining possible error bars is specially done for **q**_5_ and **q**_6_ spots with the increasing size of the outer circle. The averaged values of these data are taken as the error bars for **q**_5_ and **q**_6_ spots and shown in **b**. **d** The average of the intensities for each scattering spots measured in four areas of three samples at ±20 meV. The error bars in **d** are calculated through the standard deviations from the statistics. Note the error bars in **b** and **d** have different meanings. Those in **b** give the uncertainty of the PR-QPI intensity calculated for a particular scattering spot in one FOV. Those in **d** reflect the uncertainty among different FOVs and samples

Concerning the error bars on the PR-QPI intensities, in Fig. 6 we illustrate the statistical results measured on three samples in four different areas at ±20 meV and add the error bars to the related figures. For the measurement in one field of view (FOV) as shown in Fig. 5g–i, it has no doubt for the signs of the PR-QPI signal for **q**_1_ and **q**_7_ spots. Since the octet model is widely accepted in cuprates, our data for **q**_1_ and **q**_7_ undoubtedly show the sign change of a *d*-wave model. For other spots with weaker intensities, such as **q**_5_ and **q**_6_, we calculate the averaged intensity of the PR-QPI signal within the two circles by gradually increasing the radius of the outer circle. One can see that the averaged value outside the inner circle (shown in Fig. 6c) is rather stable versus the calculated circle size, and we thus take the average value of those data as the error bar for this particular spot. In this way we did calculations for **q**_5_ and **q**_6_ spots. One can see that the sign keeps unchanged even considering the error bars. For four different FOVs measured at ±20 meV as shown in Fig. 5 and Supplementary Figs. 6 and 7, we take average of the intensities for each spot. The corresponding error bar is calculated through the standard deviation defined as ${\mathrm{\Delta }}I = \sqrt {\mathop {\sum}\nolimits_{i = 1}^4 {\left( {I_i - \bar I} \right)^2/4} }$, with $\bar I$ the averaged intensity of one particular spot in four FOVs. The results are presented in Fig. 6d. It is clear that the error bars are all smaller than the intensities of the corresponding spots.

From above analyses we are confident that the obtained sign of the phase-referenced signal will not change for the related spots even considering the error bars. For some spots, such as **q**_3_ and **q**_4_, the phase-referenced intensity are really very weak. The major reason for this is that the scattering intensities themselves are very weak as shown in Fig. 3. Such weak intensity at large q-vector may arise from following three reasons. First, the STM tip is not infinitely sharp, then the high-*q* signals with faster oscillations in real space cannot be completely resolved. Second, the spatial variation of the wave function (Wannier function) may further suppress the high-*q* signals^41^. Third, our measurements are done in areas with finite size, which leads to the weaker intensity for large-*q* scattering.

We should point out that there are some differences of the PR-QPI intensity for different scattering spots between theoretical calculations and experimental data, although the signs are consistent each other. We realize that the theoretical calculation here can only serve as a qualitative interpretation. The shapes of the simulated FT-QPI patterns in Supplementary Figs. 2–4 are clearly different from the measured results, for example, the sizes of the scattering spots cannot be well determined from the theoretical calculations since the FT-QPI intensity shows some kind of continuing evolution in the momentum space. Actually, the Fermi surface in cuprates is very complex, and the theoretical model based on a single tight-binding band structure can only capture some features of the QPI results. Furthermore, the cuprate systems have strong correlation effect, and the Fermi arcs/pockets observed by experiment cannot be well understood by the band theory. Thus, it is reasonable to have discrepancy between experiment and theory concerning the intensity and the size of the FT-QPI spots.

## Discussion

Although consistency has been found between the experimental data and theoretical calculations by using the DBS-QPI technique, however, one may argue that the original phase-referenced QPI method^19,20^ was specially designed for the case of a single impurity. In the case of Bi-2212, there may be many randomly distributed weak impurities which prevent us from finding an area with a well-isolated impurity. As a result, we can only measure in a large area with multiple impurities with a complex pattern of standing waves in the conductance mappings as shown in Fig. 3a–f. Our primary concern is whether the phase message of the order parameter can be effectively extracted in the case of multiple impurities.

The initial theoretical work of the DBS-QPI technique^19^ has actually provided a treatment for the multi-impurity system by applying the phase correction^42^ to the measured FT-QPI pattern for multiple impurities *g*_m_(**q**, *E*). The FT-QPI for a single impurity can be derived as

4 $$g_{\mathrm{s}}\left( {{\mathbf{q}},E} \right) = \frac{{g_{\mathrm{m}}\left( {{\mathbf{q}},E} \right)}}{{C({\mathbf{q}})}} = \frac{{|g_{\mathrm{m}}({\mathbf{q}},E)|}}{{|C({\mathbf{q}})|}}e^{i\left[ {\varphi _{\mathrm{m}}\left( {{\mathbf{q}},E} \right) - \varphi _{\mathrm{C}}\left( {\mathbf{q}} \right)} \right]}.$$

Here $C\left( {\mathbf{q}} \right) = \mathop {\sum}\nolimits_j {e^{ - i{\mathbf{q}} \cdot {\mathbf{R}}_j}}$ is the correction term with **R**_*j*_ the location of the *j*th impurity. The subscripts s, m, and C represent the cases for single impurity, multi-impurity, and correction term, respectively. The *φ*_m_(**q**, *E*) and *φ*_c_(**q**) are the phases of *g*_m_(**q**, *E*) and *C*(**q**), respectively. The prerequisites for using this formula are assuming identical scattering potentials for all impurities and no interaction among them. The PR-QPI signal for a single impurity can be obtained by applying Eqs. (2) and (3) to the corrected FT-QPI *g*_m_(**q**, *E*), see Eq. (4). From the QPI images shown in Fig. 3, one can clearly see that there should be many weak impurities on the surface and it is very difficult to determine the exact coordinates of these impurities. Considering the fact that the correction factor $C\left( {\mathbf{q}} \right) = \mathop {\sum}\nolimits_j {e^{ - i{\mathbf{q}} \cdot {\mathbf{R}}_j}}$ is energy independent and yields a common phase shift for both positive and negative energy, the phase for single impurity after correction can be written as *φ*_s_(**q**, ±*E*) = *φ*_m_(**q**, ±*E*) − *φ*_*C*_(**q**), and the phase difference *φ*_s_(**q**, −*E*) − *φ*_s_(**q**, +*E*) = *φ*_m_(**q**, −*E*)−*φ*_m_(**q**, +*E*) remains unchanged before and after correction. It means that the sign of PR-QPI signal at the negative energy for multiple impurities will be exactly the same as the one for a single impurity.

For this issue we can also get support from the theoretical calculations. To illustrate that, we calculated the results for 60 impurities randomly distributed on the surface with the same scattering potential *V*_s_ = 20 meV, and also for 60, 100, and 500 randomly distributed impurities with the random scattering potential values from 0 to 100 meV. The results are shown in Supplementary Fig. 8. We want to emphasize that we have already taken the interference between impurities into account in the calculation, in other words the result of the multi-impurity is not the linear summation of the effect arising from individual impurities. Our simulation results show that the PR-QPI signal for each particular scattering spot (Supplementary Fig. 8) under different multi-impurity situations mentioned above has the same sign but different value compared with the situation of the single impurity (Supplementary Figs. 2–4). We have repeated each kind of simulation for 100 times with different distributions of the impurities, and can obtain the same conclusion. Hence, this phase-sensitive method for multi-impurities can provide us the same message as for a single impurity.

As presented above, we use the multi-DBS-QPI method to prove the superconducting gap reversal in optimally doped Bi-2212, which is consistent very well with the *d*-wave gap structure. However, with these results, one may argue that perhaps the sign-preserved gap also gives rise to such changes of the phase difference between the positive and negative energies. It has been calculated and argued that, the scattering from a non-magnetic impurity can barely induce an impurity bound state in an isotropic-*s*-wave superconductor^43^. If the gap is highly anisotropic, or say its value varies in a wide range, there may be some impurity induced bound states even if the superconducting gap is nodeless. We thus carry out further simulations for the superconductors with different gap functions, for examples, a nodal but sign-preserved gap ${\it{\Delta}} \left( {\mathbf{k}} \right) = 23|\,{\mathrm{cos}}\,k_x - {\mathrm{cos}}\,k_y| \,({\mathrm{meV}})$ and a nodeless gap ${\it{\Delta}}\left( {\mathbf{k}} \right) = 23\left| {{\mathrm{cos}}\,k_x - {\mathrm{cos}}\,k_y} \right| + 2 \, ({\mathrm{meV}})$. The same scattering scalar potential *V*_s_ = 20 meV is used for the non-magnetic impurity. The resultant tunneling spectra for above two gaps are shown in Supplementary Figs. 9a and 10a, respectively. One can see that the non-magnetic impurities only slightly shift the position of the coherence-peaks, and have negligible influence on the LDOS near zero bias. We also calculate the PR-QPI images for the single and multi-impurity situation with different forms of the sign-preserved superconducting gaps mentioned above, and the related simulations are shown in Supplementary Figs. 9 and 10. One can see that the results for the multi-impurity situation are always similar to the case for a single impurity. Furthermore, all the PR-QPI signals for seven characteristic scattering spots are positive in this case. Therefore, we exclude the possibility of the sign-preserved gap in Bi-2212.

Another argument concerning our conclusion may be that the impurities could be magnetic ones instead of non-magnetic ones. The calculation for the magnetic scattering should be carried out and compared with experiment. This is actually not relevant for the optimally doped Bi-2212 since, as far as we know, no magnetic impurities with even moderate scattering potentials have been reported in literatures^44^. We further corroborate this point with two more basic arguments. (1) If the magnetic impurities with certain scattering potential are present, we would have seen some strong resonant state peaks within the gap. This has not been observed in such samples. (2) We have also done the calculations for superconductors with a *d*-wave gap with magnetic impurities, and find that the PR-QPI signals for seven characteristic scattering spots are of the same signs when the magnetic scattering potential (*V*_m_) is smaller than 200 meV, see Supplementary Fig. 11. By using the DBS-QPI method, we clearly prove the sign-changing *d*-wave pairing symmetry in optimally doped Bi-2212. Our experiments and analyzing technique suggest that this method may also be applicable to other unconventional superconductors if the gap has a sign change. This will provide a more easily accessible way to determine the gap structure of unconventional superconductors.

## Methods

### Sample synthesis and characterization

Optimally doped Bi_2_Sr_2_CaCu_2_O_8+δ_ single crystals were grown by the floating-zone technique^45^. The quality of the sample has been checked by the DC magnetization measurement before the STM measurements. The critical temperature *T*_c_ is about 90 K as determined from the DC magnetization measurement.

### STM/STS measurements

The STM/STS measurements were done in a scanning tunneling microscope (USM-1300, Unisoku Co., Ltd.) with ultra-high vacuum, low temperature, and high magnetic field. The Bi-2212 samples were cleaved at room temperature in an ultra-high vacuum with a base pressure of about 1 × 10^−10^ torr. The electrochemically etched tungsten tips or the Pt/Ir alloy tips were used for all the STM/STS measurements. A lock-in technique was used for measuring tunneling spectrum with an ac modulation of 1.5 mV and 987.5 Hz. All the data were taken at 1.5 K. The QPI images were measured with the resolution of 256 pixels × 256 pixels. The set-point condition was *V*_set_ = −100 mV, *I*_set_ = 100 pA. QPI images were measured individually for each pair of positive and negative energies ±*E*, and each scanning took about 2000 min. The FT-QPI patterns were corrected by using the Lawler–Fujita algorithm^25^ to reduce the distortions from the non-orthogonality in the *x*/*y* axes. This correction can raise the signal-to-noise ratio of FT-QPI patterns and it should not give influence on the final determination of the sign of the PR-QPI signal corresponding to each primary scattering spot. The presented FT-QPI and PR-QPI patterns were mirror-symmetrized to reduce the noise. When treating the QPI data of sample 2, we have removed an unphysical background signal showing as two vertical lines appearing symmetrically around *q*_*x*_ = 0. This will not give influence on the final results of PR-QPI.

### Theoretical calculations

We have employed a single tight-binding band structure similar to the one proposed in the previous report^46^, with the energy dispersion given by

5 $$\begin{array}{*{20}{l}} {\varepsilon _k} \hfill & = \hfill & { - 2t_1\left( {{\mathrm{cos}}\,k_x + {\mathrm{cos}}\,k_y} \right) + 4t_2\,{\mathrm{cos}}\,k_x\,{\mathrm{cos}}\,k_y - 2t_3\left( {{\mathrm{cos}}\,2k_x} \right.} \hfill \\ {} \hfill & {} \hfill & {\left. { {}+ {\mathrm{cos}}\,2k_y} \right) - 2t_4({\mathrm{cos}}\,2k_x\,{\mathrm{cos}}\,k_y + \,{\mathrm{cos}}\,k_x\,{\mathrm{cos}}\,2k_y)-\mu .} \hfill \end{array}$$

The parameters (*t*_1_, *t*_2_, *t*_3_, *t*_4_, *μ*) = (100, 36, 10, 1.5, −155) are used in the calculations with the units of meV. Using a standard T-matrix method^31^, we simulate the LDOS around a single impurity and for the case of multiple impurities. In Nambu space, the BCS Hamiltonian is given by

6 $$H_0 = \left( {\begin{array}{*{20}{c}} {\varepsilon_{k}} & {\it{\Delta}}_{k} \\ {\it{\Delta}}_{k}^ {\ast} & {- \varepsilon _{-k}} \end{array}} \right)$$

Suppose that we have *N* impurities located at **r**_1_, **r**_2_, **r**_3_, …, and **r**_*N*_ (Set *N* = 1 reduced to the case of a single impurity). The Green’s function in real space^47^ can be formulated by

7 $$G\left( {{\mathbf{r}},{\mathbf{r}}\prime ,E} \right) = G_0\left( {{\mathbf{r}},{\mathbf{r}}\prime ,E} \right) + \mathop {\sum}\limits_{i,j} {G_0({\mathbf{r}},{\mathbf{r}}_i,E)T({\mathbf{r}}_i,{\mathbf{r}}_j,E)G_0({\mathbf{r}}_j,{\mathbf{r}}\prime ,E)} ,$$

where $G_0\left( {{\mathbf{r}},{\mathbf{r}}\prime ,E} \right) = \frac{1}{M}\mathop {\sum}\nolimits_{\boldsymbol{k}} {G_0\left( {{\boldsymbol{k}},E} \right)e^{i{\boldsymbol{k}} \cdot ({\mathbf{r}} - {\mathbf{r}}\prime )}}$ with *M* the numbers of unit cells, *G*_0_(**k**, *E*) the unperturbed Green’s function in reciprocal space and the many-impurity 2*N* × 2*N* T-matrix is determined by

8 $${\mathbf{T}}^{-1} = {\mathbf{V}}^{-1} - {\mathbf{G}}_0,$$

where

9 $${\mathbf{G}}_{\mathbf{0}} = \left( {\begin{array}{*{20}{c}} {G_0({\mathbf{r}}_1,{\mathbf{r}}_1,E)} & \cdots & {G_0({\mathbf{r}}_1,{\mathbf{r}}_N,E)} \\ \vdots & \ddots & \vdots \\ {G_0({\mathbf{r}}_N,{\mathbf{r}}_1,E)} & \cdots & {G_0({\mathbf{r}}_N,{\mathbf{r}}_N,E)} \end{array}} \right),$$

10 $${\mathbf{V}} = \left( {\begin{array}{*{20}{l}} {V_1} \hfill & 0 \hfill & \cdots \hfill & 0 \hfill \\ 0 \hfill & {V_2} \hfill & \cdots \hfill & 0 \hfill \\ \vdots \hfill & \vdots \hfill & \ddots \hfill & \vdots \hfill \\ 0 \hfill & 0 \hfill & \cdots \hfill & {V_N} \hfill \end{array}} \right).$$

Here, *V*_*i*_ = *V*_s_*τ*_3_ + *V*_m_*τ*_0_, with *V*_s_ the scalar potential, *V*_m_ the magnetic scattering potential. *V*_m_ = 0 for non-magnetic impurities. Then we can obtain the spin-summed LDOS given by

11 $$g\left( {{\mathbf{r}},E} \right) = - \frac{1}{{\mathrm{\pi }}}{\mathrm{Im}}\left[ {\frac{{\tau _0 + \tau _3}}{2}G\left( {{\mathbf{r}},{\mathbf{r}},E} \right) + \frac{{\tau _0 - \tau _3}}{2}G\left( {{\mathbf{r}},{\mathbf{r}}, - E} \right)} \right],$$

where *τ*_*i*_ is the Pauli matrix spanning Nambu space. Referring to Eqs. (2) and (3), we can get the simulated PR-QPI images as shown in Supplementary Figs. 2–4 and 8–11. For the case of multiple impurities, we randomly assign the impurities to the lattice sites in our simulated FOV either with the identical scattering potential or with the randomly distributed scattering potentials within 100 meV.

## Supplementary information

Supplementary Information

## Data Availability

The data that support the plots within this paper and other findings of this study are available from the corresponding author upon reasonable request.
