# Supplementary material for: Directly visualizing the sign change of d-wave superconducting gap in Bi2Sr2CaCu2O8+δ by phase-referenced quasiparticle interference
Source: Nat Commun. 2019 Apr 8;10:1603. doi: 10.1038/s41467-019-09340-5 (PMC6453940; doi:10.1038/s41467-019-09340-5)
Supplement: Supplementary file 1 — Supplementary Information [file 41467_2019_9340_MOESM1_ESM.pdf]

Supplementary Information

Directly visualizing the sign change of *d*-wave superconducting gap in $Bi_2Sr_2CaCu_2O_{8+\delta}$ by phase-referenced quasiparticle interference

Qiangqiang Gu, et al.

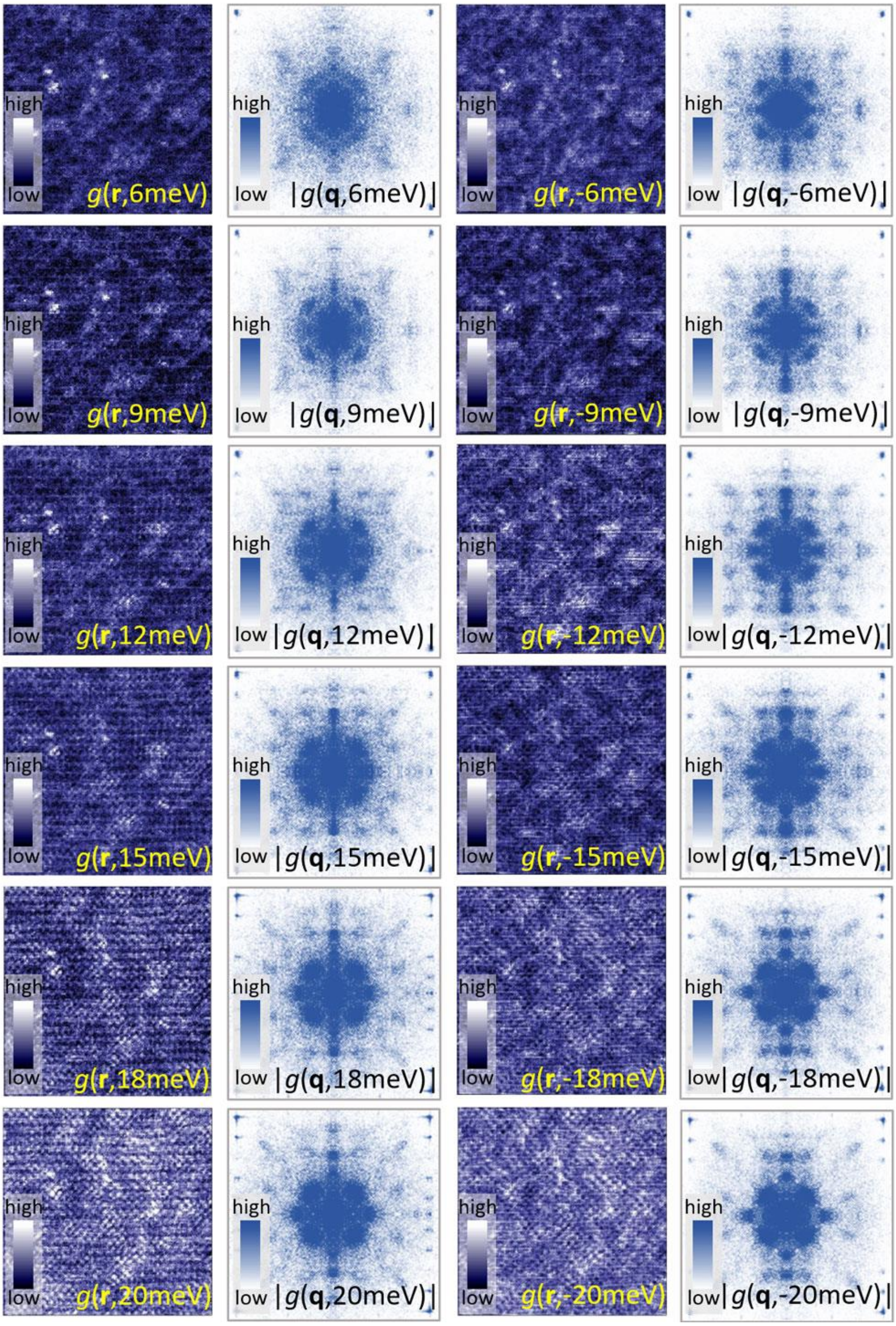

Supplementary Figure 1 Measured QPI images and corresponding FT-QPI patterns at different energies on Bi-2212. V_{set} = −100 mV, I_{set} = 100 pA.

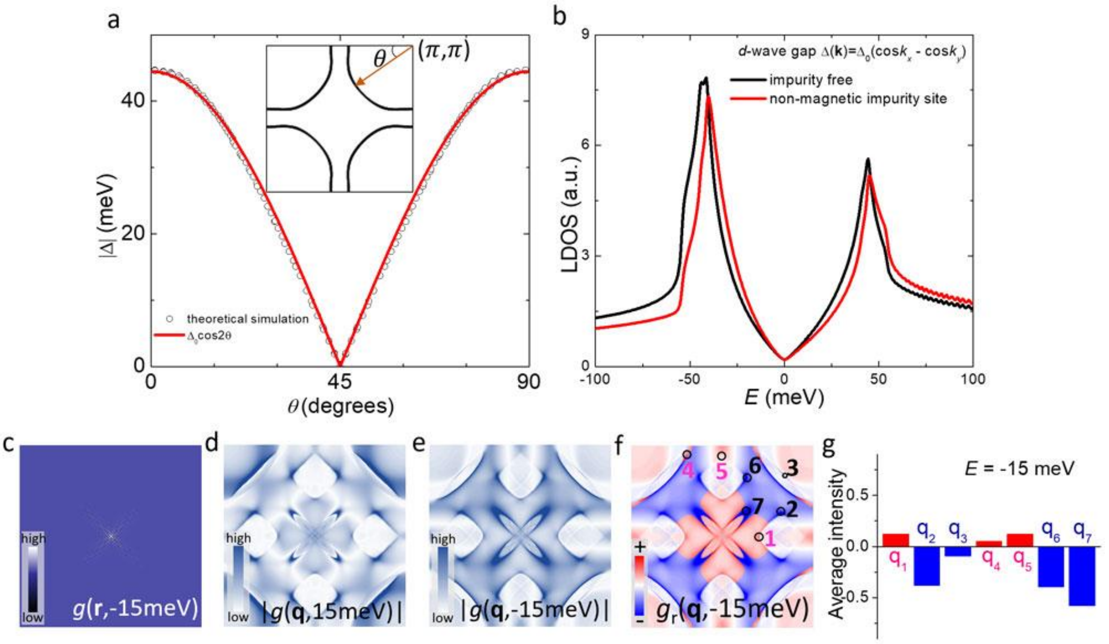

Supplementary Figure 2 Theoretical calculated tunneling spectra and PR-QPI results in a *d*-wave superconductor. **a** Fermi surface and gap function calculated for a *d*-wave superconductor. The inset in **a** shows the calculated Fermi surface and the definition of the parameter angle θ. The *d*-wave gap function $\Delta(\mathbf{k}) = \Delta_0(\cos k_x - \cos k_y)$ with $\Delta_0 = 23$ meV is assigned to the Fermi surface, and the resultant angle θ dependent superconducting gap along the Fermi surface is shown in **a**. The gap function along the Fermi surface (open symbols) is consistent with a *d*-wave function $\Delta(\theta) = 44.5 \cos 2\theta$ meV. **b** The tunneling spectra in an impurity-free area and on site of the non-magnetic impurity. The scattering scalar potential is set to $V_s = 20$ meV for the non-magnetic impurity. **c** Simulated LDOS at −15 meV around the single non-magnetic impurity with dimensions of 512×512 atom lattice. The impurity is set to be at the center of the image. **d**,**e** FT-QPI patterns for the single impurity at ± 15 meV. **f** The resultant PR-QPI image $g_r(\mathbf{q}, -15\text{ meV})$ calculated by DBS-QPI method from the simulation results in **d** and **e**. **g** Average intensity per pixel for the characteristic scattering spots in **f**. One can find that the $g_r(\mathbf{q}, -15\text{ meV})$ values near gap-sign-preserved scattering vectors $\mathbf{q}_1$, $\mathbf{q}_4$, and $\mathbf{q}_5$ are positive, while the values near gap-sign-reversed scattering vectors $\mathbf{q}_2$, $\mathbf{q}_3$, $\mathbf{q}_6$, and $\mathbf{q}_7$ are negative.

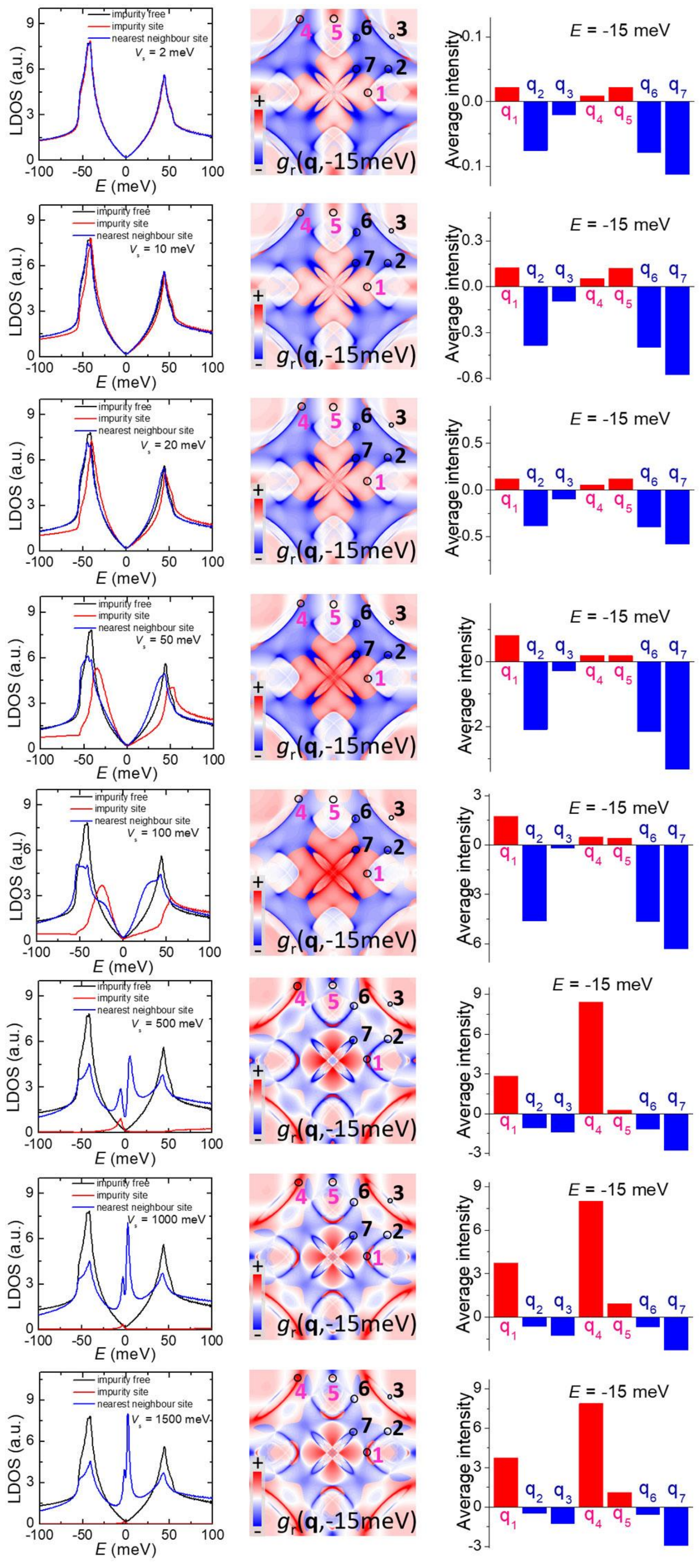

Supplementary Figure 3 Simulated results around a single non-magnetic impurity with different positive scattering potentials in a *d*-wave superconductor.

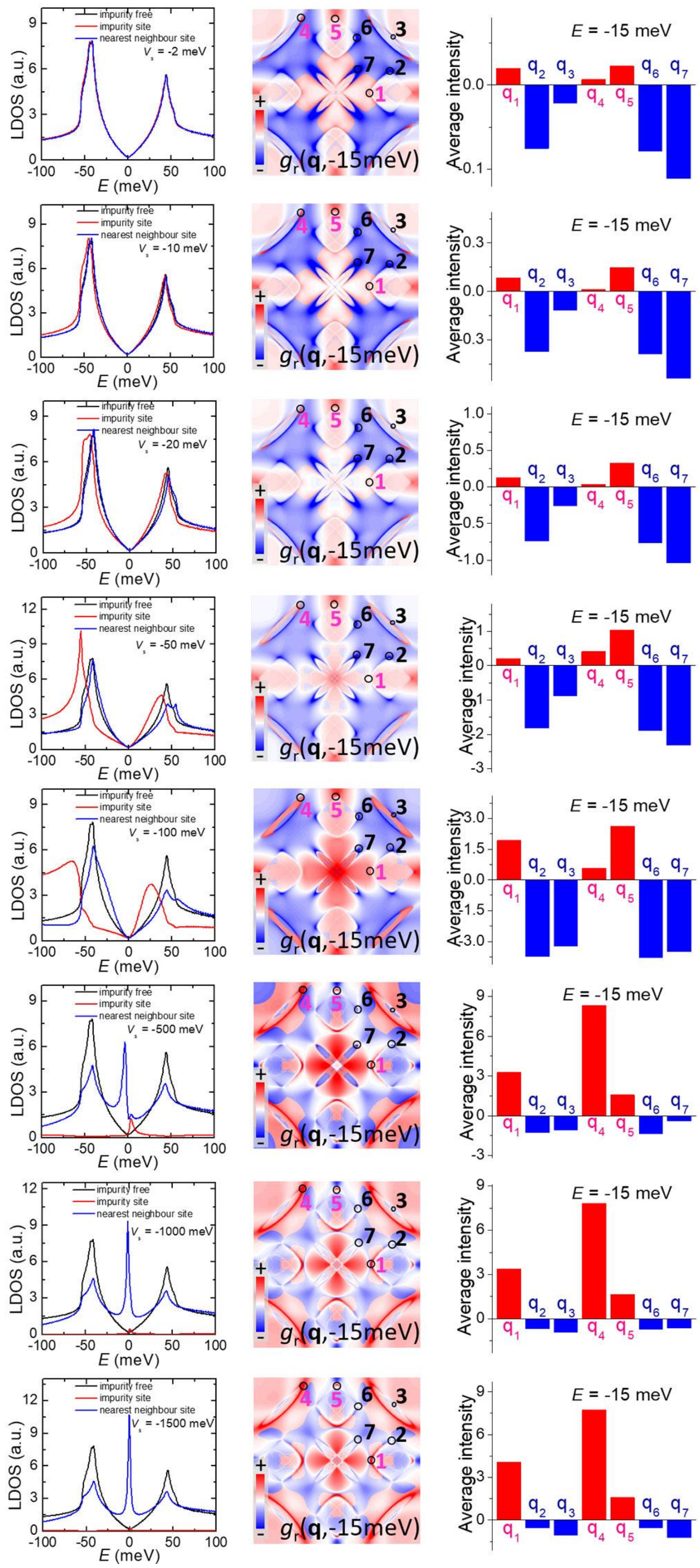

Supplementary Figure 4 Simulated results around a single non-magnetic impurity with different negative scattering potentials in a *d*-wave superconductor.

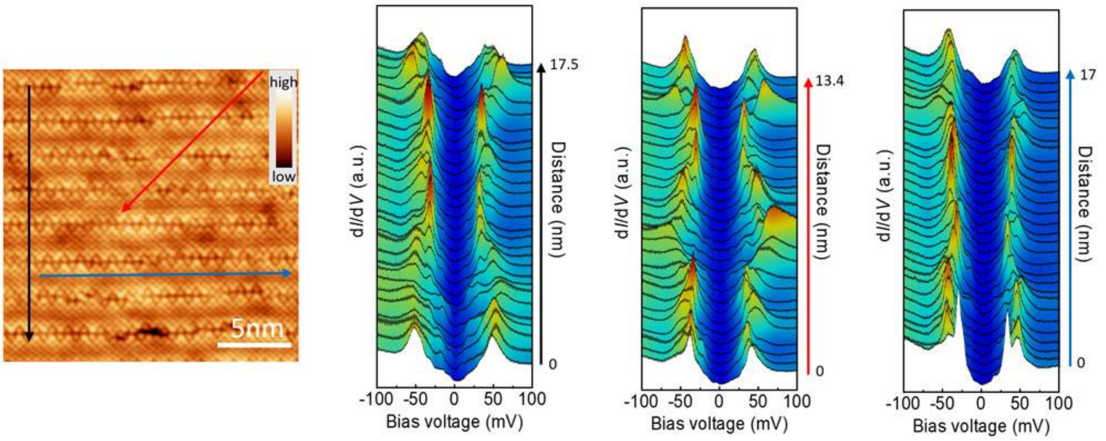

Supplementary Figure 5 Topographic image (V_{bias} = −200 mV, I_t = 50 pA) and tunneling spectra measured on Bi-2212. The spectra are measured along different arrowed lines in the topographic image.

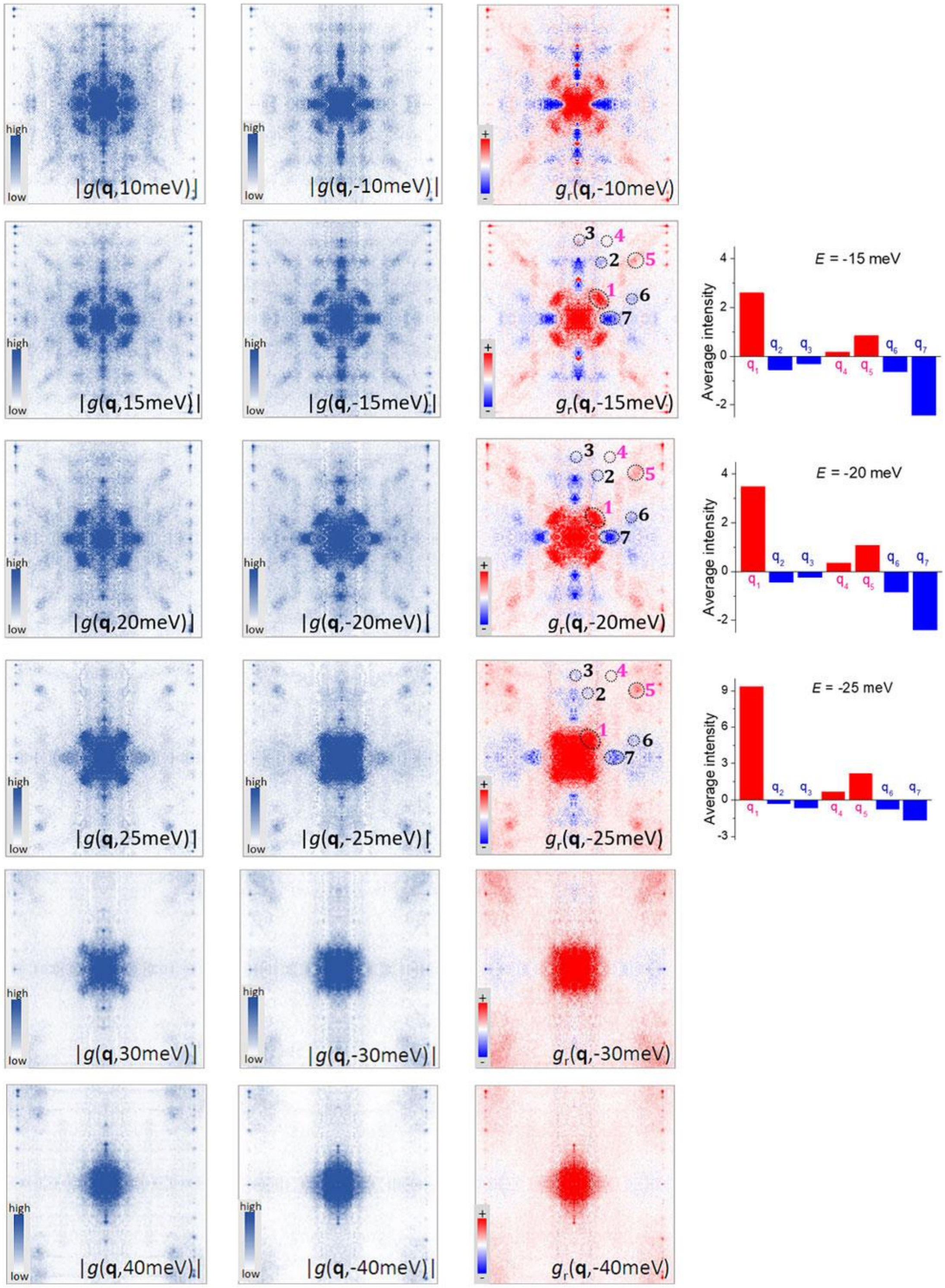

Supplementary Figure 6 Control experiments of FT-QPI patterns and corresponding PR-QPI patterns at different energies measured in area-1 on sample 2. The left two columns show the FT-QPI intensity maps. The 3rd column shows the PR-QPI patterns out of the corresponding data. The 4th column shows the PR-QPI intensity of the seven characteristic spots derived from the 3rd column. The PR-QPI pattern at -20 meV is the same as the one in Fig. 6a. $V_{set} = -100$ mV, $I_{set} = 100$ pA.

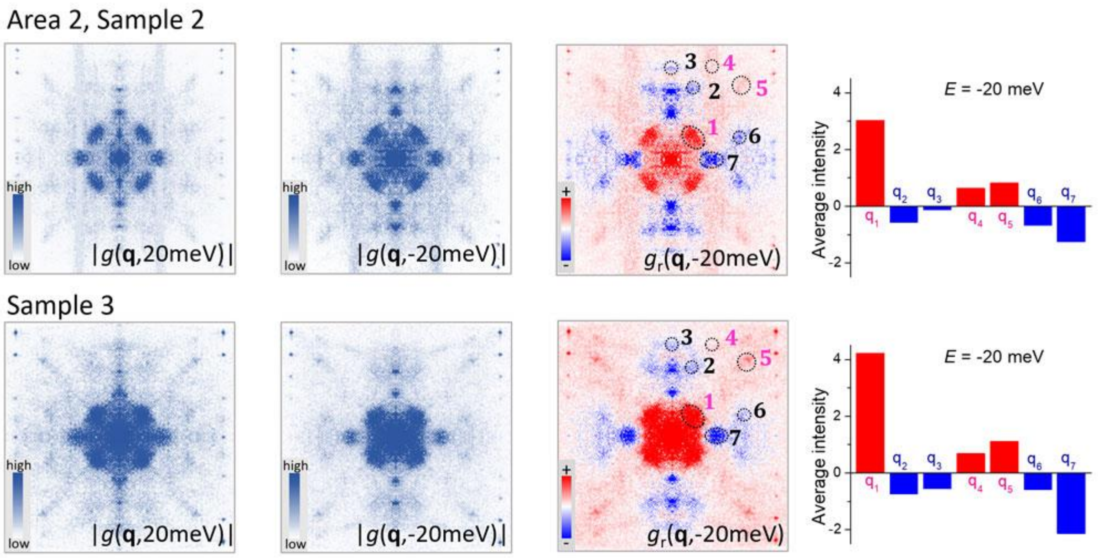

Supplementary Figure 7 Control experiments of FT-QPI patterns and corresponding PR-QPI patterns at ±20 meV measured in area-2 of sample 2 and in an area of sample 3. The left two columns show the FT-QPI intensity maps. The 3rd column shows the PR-QPI patterns out of the corresponding data. The 4th column shows the PR-QPI intensity of the seven characteristic spots derived from the 3rd column. V_{set} = −100 mV, I_{set} = 100 pA.

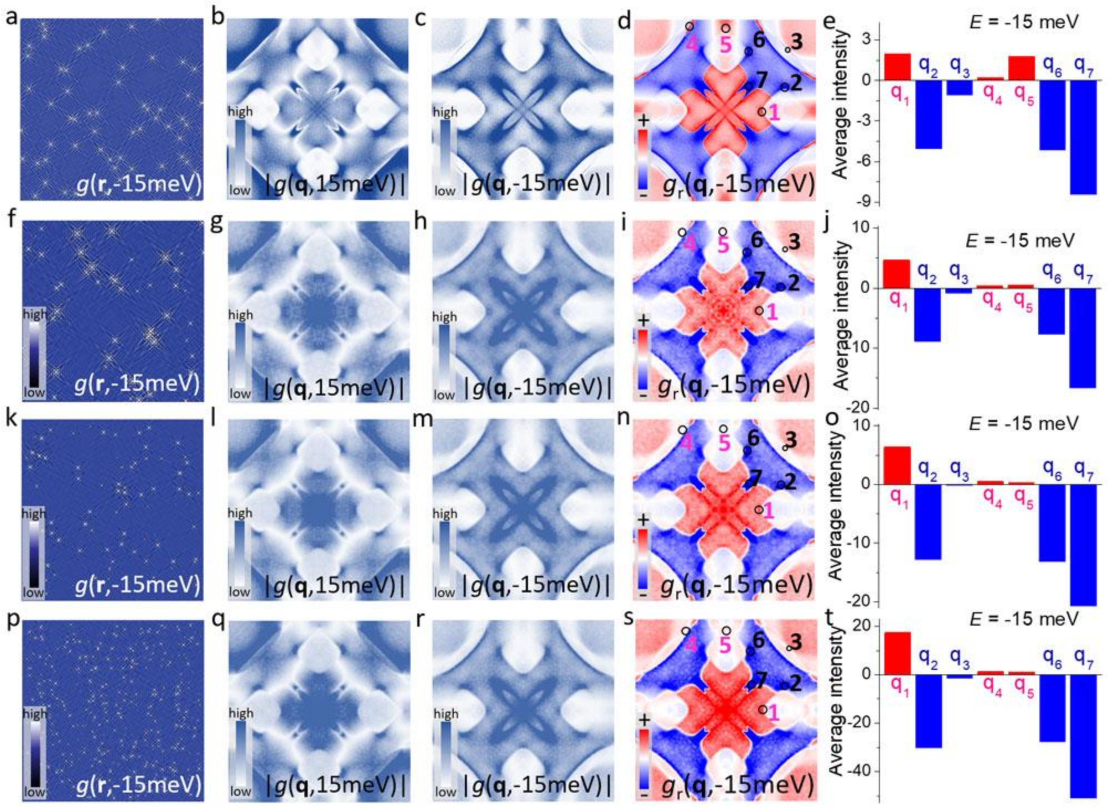

Supplementary Figure 8 Theoretical simulated PR-QPI results for the case of multiple non-magnetic impurities with a *d*-wave superconducting gap function. **a**-**e** Simulation results for the case of 60 same impurities with same scattering potential $V_{\mathrm{s}} = 20$ meV. These impurities are randomly distributed in an area with dimensions of 512×512 atom lattice as shown in **a**. **f**-**t** Simulation results for the cases of 60 (**f**-**j**), 100 (**k**-**o**) and 500 (**p**-**t**) randomly distributed impurities with the random scattering potential values from 0 to 100 meV. The areas are also with dimensions of 400×400 atom lattice. One can see that the signs of PR-QPI signal for the characteristic scattering spots are the same as the ones of the single impurity situation.

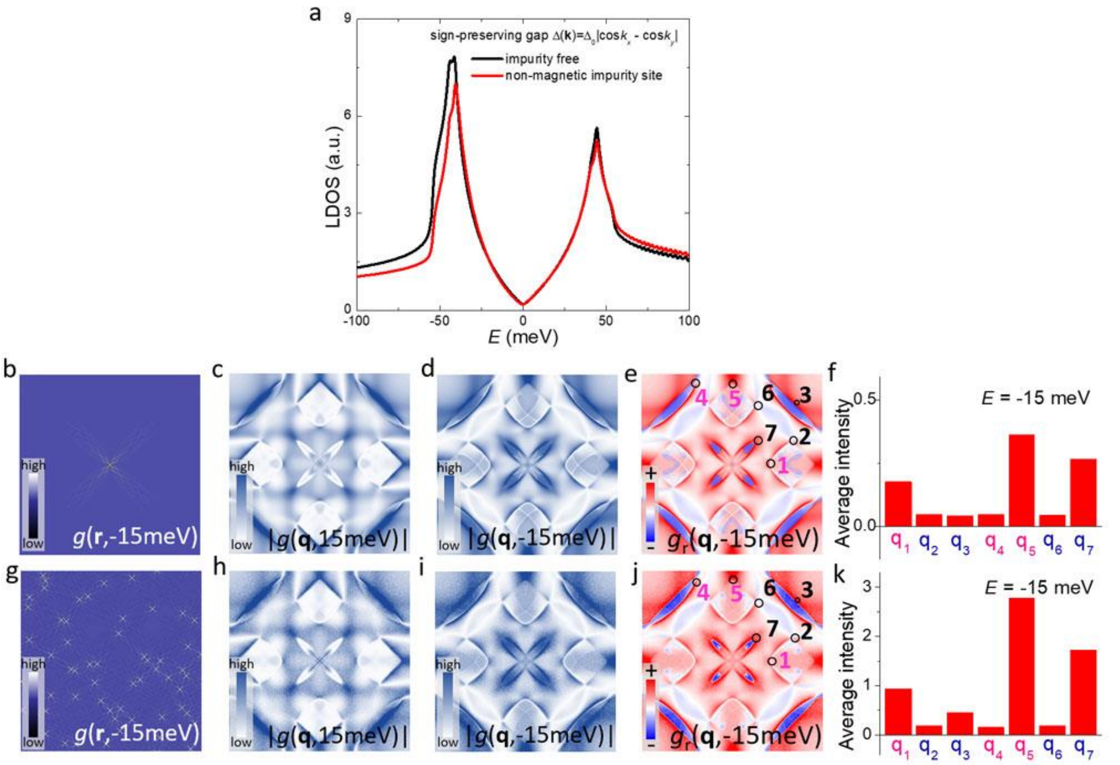

Supplementary Figure 9 Theoretical simulated PR-QPI results with a sign-preserved nodal gap function. **a** The tunneling spectra in an impurity-free area and on site of the non-magnetic impurity for a superconductor with a nodal but sign-preserved gap. The Fermi surface is the same as the one in Supplementary Figure 2, and the superconducting gap function used in the calculation is $\Delta(\mathbf{k}) = \Delta_0|(\cos k_x - \cos k_y)|$ with $\Delta_0 = 23$ meV. The scattering scalar potential is set to $V_s = 20$ meV for the non-magnetic impurity. **b** Simulated LDOS around a single non-magnetic impurity at −15 meV with dimensions of 512×512 atom lattice. The impurity is set to be at the center of the image. **c**,**d** FT-QPI patterns at $\pm$15 meV for the single impurity. **e** The resultant PR-QPI image g_r(**q**, −15 meV) calculated by DBS-QPI method from the simulation results in **c** and **d**. **f** Average intensity per pixel for the characteristic scattering spots in **e**. One can see that the g_r(**q**, −15 meV) values near all characteristic scattering vectors are positive. **g**-**k** The corresponding simulation results for the case of multiple impurities. The 60 impurities are the same as the one in **b**, and they are randomly distributed in an area with dimensions of 512×512 atom lattice as shown in **g**. The locations of impurities are the same as the ones in Supplementary Figure 8a.

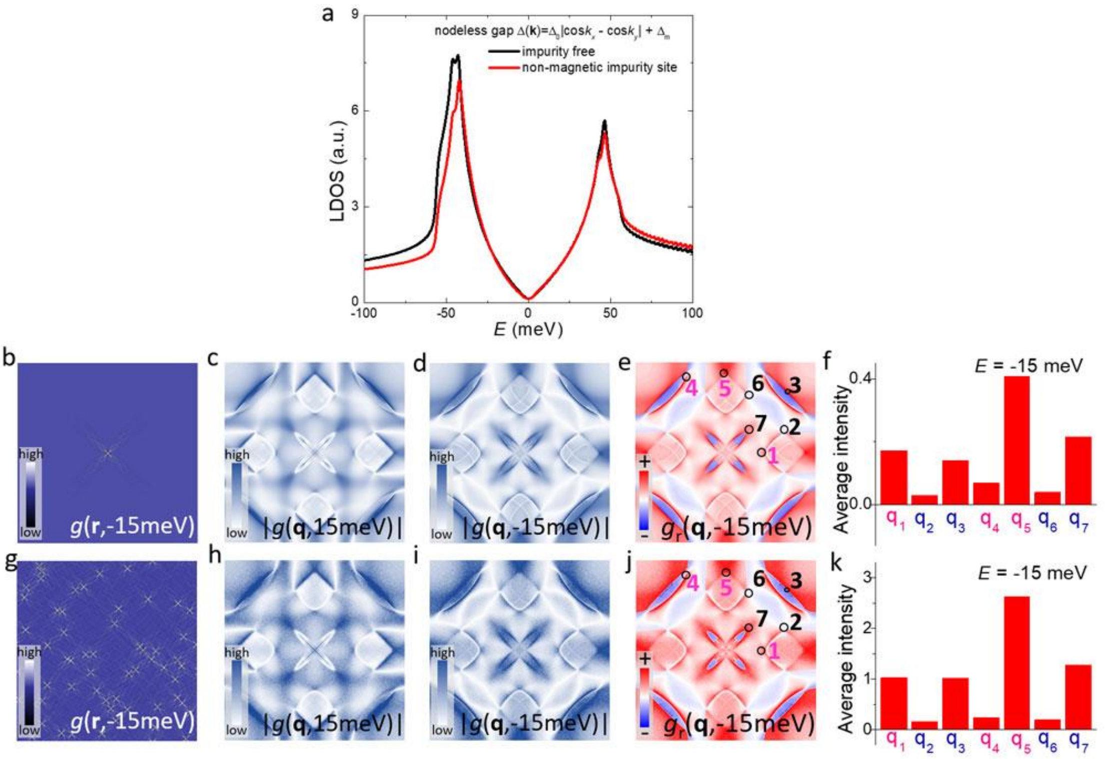

Supplementary Figure 10 Theoretical simulated PR-QPI results with a sign-preserved nodeless gap function. **a** The tunneling spectra in an impurity-free area and on site of the non-magnetic impurity for a superconductor with a sign-preserved nodeless gap. The Fermi surface is the same as the one in Supplementary Figure 2, and the superconducting gap function used in the calculation is $\Delta(\mathbf{k}) = \Delta_0\left|\cos k_x - \cos k_y\right| + \Delta_\mathrm{m}$ with $\Delta_0 = 23$ meV, $\Delta_\mathrm{m} = 2$ meV. The scattering scalar potential is set to $V_s = 20$ meV for the non-magnetic impurity. **b** Simulated LDOS around a single non-magnetic impurity at −15 meV with dimensions of 60×60 atom lattice. The impurity is set to be at the center of the image. **c**,**d** FT-QPI patterns for the single impurity at $\pm$15 meV. **e** The resultant PR-QPI image g_r(**q**, −15 meV) calculated by DBS-QPI method from the simulation results in **c** and **d**. **f** Average intensity per pixel for the characteristic scattering spots in **e**. One can see that the g_r(**q**, −15 meV) values near all characteristic scattering vectors are positive. **g**-**k** The corresponding simulation results for the case of multiple impurities. The 60 impurities are the same as the one in **a**, and they are randomly distributed in an area with dimensions of 512×512 atom lattice as shown in **g**. The locations of impurities are the same as the ones in Supplementary Figure 8a.

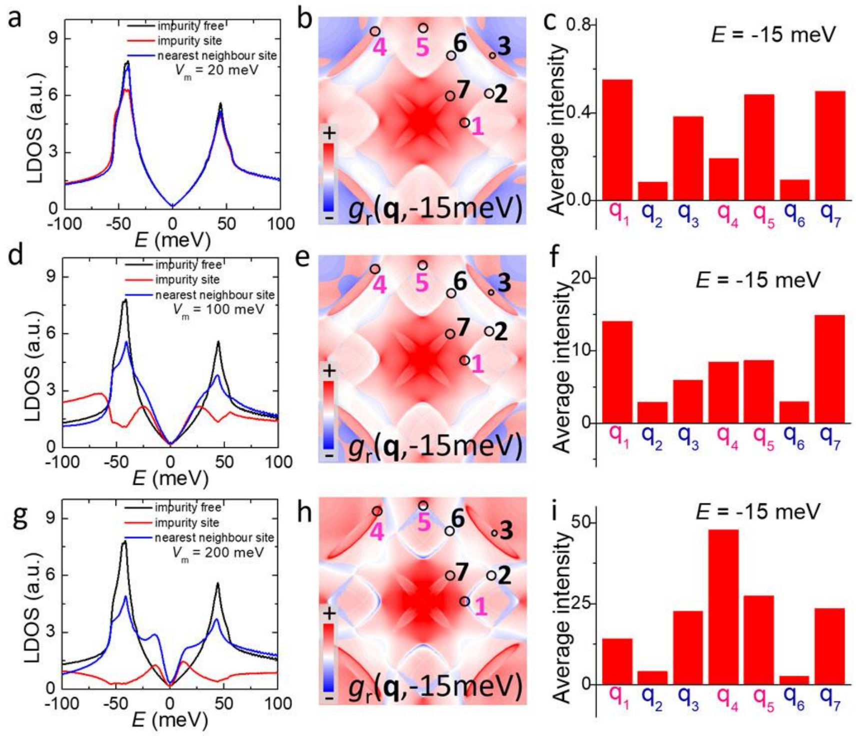

Supplementary Figure 11 Simulated results around a single magnetic impurity with different magnetic scattering potentials in a *d*-wave superconductor.
